# A two-step transport pathway allows the mother cell to nurture the developing spore in *Bacillus subtilis*

**DOI:** 10.1371/journal.pgen.1007015

**Published:** 2017-09-25

**Authors:** Fernando H. Ramírez-Guadiana, Alexander J. Meeske, Christopher D. A. Rodrigues, Rocío del Carmen Barajas-Ornelas, Andrew C. Kruse, David Z. Rudner

**Affiliations:** 1 Department of Microbiology and Immunobiology, Harvard Medical School, Boston, MA, United States of America; 2 Department of Biochemistry and Molecular Pharmacology, Harvard Medical School, Boston, MA, United States of America; Indiana University, UNITED STATES

## Abstract

One of the hallmarks of bacterial endospore formation is the accumulation of high concentrations of pyridine-2,6-dicarboxylic acid (dipicolinic acid or DPA) in the developing spore. This small molecule comprises 5–15% of the dry weight of dormant spores and plays a central role in resistance to both wet heat and desiccation. DPA is synthesized in the mother cell at a late stage in sporulation and must be translocated across two membranes (the inner and outer forespore membranes) that separate the mother cell and forespore. The enzymes that synthesize DPA and the proteins required to translocate it across the inner forespore membrane were identified over two decades ago but the factors that transport DPA across the outer forespore membrane have remained mysterious. Here, we report that SpoVV (formerly YlbJ) is the missing DPA transporter. SpoVV is produced in the mother cell during the morphological process of engulfment and specifically localizes in the outer forespore membrane. Sporulating cells lacking SpoVV produce spores with low levels of DPA and cells engineered to express SpoVV and the DPA synthase during vegetative growth accumulate high levels of DPA in the culture medium. SpoVV resembles concentrative nucleoside transporters and mutagenesis of residues predicted to form the substrate-binding pocket supports the idea that SpoVV has a similar structure and could therefore function similarly. These findings provide a simple two-step transport mechanism by which the mother cell nurtures the developing spore. DPA produced in the mother cell is first translocated into the intermembrane space by SpoVV and is then imported into the forespore by the SpoVA complex. This pathway is likely to be broadly conserved as DPA synthase, SpoVV, and SpoVA proteins can be found in virtually all endospore forming bacteria.

## Author summary

All pathogenic and non-pathogenic bacteria that differentiate into dormant endospores including *Clostridium difficile*, *Bacillus anthracis*, and *Bacillus subtilis*, contain very high concentrations of the small molecule dipicolinic acid (DPA). This molecule displaces water in the spore core where it plays an integral role in spore resistance and dormancy. DPA and its contribution to spore dehydration were discovered in 1953 but the molecular basis for its accumulation in the spore has remained unclear. The developing endospore resides within a mother cell that assembles protective layers around the spore and nurtures it by providing mother-cell-produced molecules. DPA is produced in the mother cell at a late stage in development and then must be translocated across two membranes into the spore core. Here, we report the discovery of the missing DPA transporter, homologs of which are present in virtually all endospore-forming bacteria. Our data provide evidence for a simple two-step transport pathway in which the mother cell nurtures the developing spore by sequentially moving DPA across the two membranes that surround it.

## Introduction

Endospore formation, as the name implies, involves the differentiation of a stress-resistant and dormant cell-type *within* another cell (referred to as the mother cell). The mother cell plays a central role in this process packaging the prospective spore (the forespore) in a series of protective layers and nurturing it by providing mother-cell-produced molecules (reviewed in [1–3]). There are two stages during this developmental process in which the mother cell is thought to nurture the forespore. In the first, a putative secretion apparatus or “feeding tube” is assembled across the membranes that separate the two cells but the transported molecule(s) have yet to be identified [4–6]. In the second, the transported molecule pyridine-2,6-dicarboxylic acid (dipicolinic acid or DPA) has been known for more than half a century [7] but the molecular basis for its transport and accumulation in the spore has remained incompletely understood. Here, we provide evidence for a simple two-step transport mechanism in which DPA is moved sequentially across the two membranes that separate mother cell and forespore.

In response to the onset of starvation, *B*. *subtilis* initiates the developmental process of sporulation (reviewed in [8–10]). The first landmark event in this process is the formation of a polar septum that divides the sporulating cell into a larger mother cell and a smaller forespore. Shortly after division, the mother cell membranes migrate around the forespore in a process resembling phagocytosis called engulfment. The completion of engulfment involves a membrane fission event [11, 12] that generates a cell within a cell in which the forespore is surrounded by two membranes: an inner membrane derived from the forespore and an outer membrane derived from the mother cell that is topologically distinct from the peripheral membranes that contain the two cells (called the sporangium). At this late stage the forespore prepares for dormancy in part through the production of small DNA binding proteins that compact the chromosome and protect it from irradiation and genotoxic stress; the synthesis of proteases that degrade these DNA binding proteins upon germination; and the production and assembly of germinant receptors and a DPA importer in the inner forespore membrane [13–16]. At the same time, the mother cell packages the forespore in a series of protective layers including a specialized layer of peptidoglycan between the inner and outer forespore membranes called the spore cortex and a multilayered spore coat composed of more than 70 mother-cell-produced proteins [2, 3]. Upon spore maturation, the mother cell lyses releasing the dormant spore into the environment.

This eight-hour morphological process is driven by sporulation-specific sigma factors that control developmental gene expression in a stage- and compartment-specific fashion [8, 10, 17]. The first cell-type-specific transcription factor, SigF, is activated immediately after polar division in the forespore. SigF controls early forespore gene expression but is also required for the activation of SigE in the mother cell. SigE controls the expression of many morphogenetic proteins including those responsible for engulfment. Shortly after the completion of engulfment a second forespore-specific transcription factor SigG becomes active, which in turn, is responsible for the activation of a second mother-cell specific transcription factor SigK. These late-acting transcription factors are required for the maturation of the dormant spore. SigG controls the expression of the DNA binding proteins that compact and protect the spore chromosome, the germinant receptors, and the SpoVA proteins that are responsible for DPA import [18]. SigK is responsible for the production of many of the proteins that make up the coat as well as enzymes involved in the production of a coat-associated polysaccharides and cell wall precursors used to synthesize the spore cortex [19]. SigK also controls the synthesis of SpoVFA and SpoVFB the enzymes that convert dihydroxydipicolinic acid, an intermediate in the lysine biosynthetic pathway, into DPA [20, 21]. DPA must then be transported into the spore where it accumulates to very high levels. DPA in complex with Ca^2+^ is thought to protect the spore DNA and also displace water resulting in core dehydration and increased heat resistance of macromolecules [15, 22].

It has been appreciated for over fifty years that dormant spores derived from the *Bacillales* and *Clostridiales* orders contain high concentrations of DPA [7, 23], yet the mechanism by which it is transported from the mother cell into the forespore has remained incompletely understood. Here, we identify SpoVV (formerly YlbJ) as the missing step in this transport pathway and provide evidence that it translocates DPA across the outer forespore membrane from the mother cell into the intermembrane space. SpoVV is synthesized in the mother cell under the control of SigE during the engulfment process ensuring its localization in the outer forespore membrane. Thus, DPA accumulation in the forespore involves three enzymes, a synthase (SpoVFAB) and two transporters (SpoVV and SpoVA) that are produced under the control of three distinct sporulation sigma factors at different stages in development. The spatio-temporal regulation of this transport pathway underscores how intimately the sporulation programs of gene expression are coordinated with morphogenesis.

## Results

### SpoVV (YlbJ) is required for DPA accumulation in dormant spores

**Fig 1:**
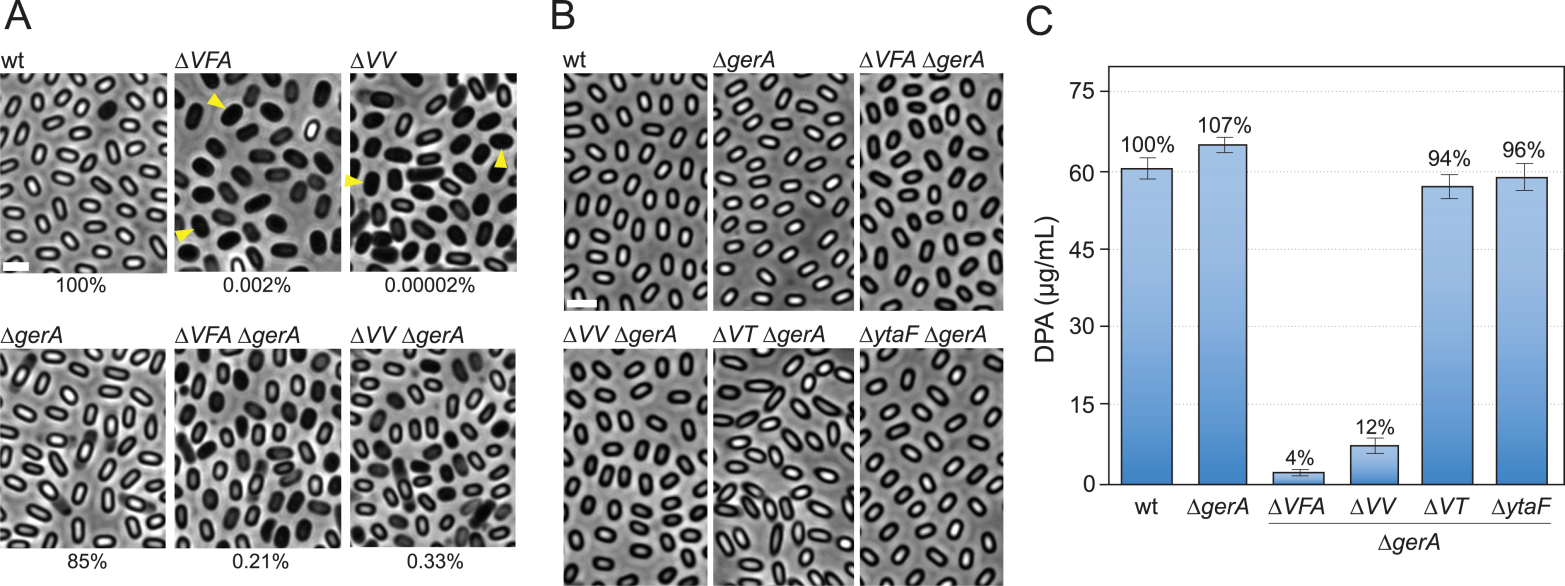
SpoVV (YlbJ) is required for DPA accumulation in dormant spores. A. Representative phase-contrast images of the indicated strains sporulated by nutrient exhaustion for 30 h at 37°C. Examples of phase-dark prematurely germinated spores are highlighted (yellow carets). Sporulation efficiencies, as determined by colony forming units after heat treatment (80°C, 20 min) are indicated below each image. Strains lacking the B subunit of the GerA receptor (GerAB) are designated ∆*gerA* for clarity. B. Representative images of purified spores used to measure DPA content. Indicated strains were sporulated by resuspension in defined minimal medium for 30 h at 37°C. Spores were purified as described in Methods. Scale bars indicate 2 μm. C. Bar graph showing DPA content in purified spores from the indicated strains. Purified spores were concentrated to an OD_600_ of 5, and incubated at 100°C for 30 min to release DPA. DPA content in the supernatant was then quantified using a colorimetric assay. Commercial DPA was used to generate a standard curve. Results shown are the average ± standard deviation of three independent biological replicates.

We recently reported that a large collection of mutations that impair the protective layers and/or the resistance properties of the developing spore inappropriately trigger the germinant receptor GerA leading to premature germination, a transition to phase-dark appearance, and a loss of wet heat resistance [24] (Fig 1A). Inactivation of this germinant receptor partially suppressed the sporulation defects associated with these mutants. Among the >25 mutants tested, the two that were most strongly suppressed in the absence of GerA were ∆*spoVFAB* and ∆*ylbJ* (Fig 1A). The *spoVFAB* operon encodes the DPA synthase that converts dihydroxydipicolinic acid into DPA [21]. Cells lacking either of these genes are reduced in sporulation efficiency by >1,000-fold as assayed by resistance to wet heat (80°C for 20 min). However, in cells lacking a functional GerA receptor, the sporulation efficiencies of the ∆*spoVFA* and ∆*spoVFB* mutants improved by more than 100-fold to 0.2% (Fig 1A and S1 Fig). Furthermore, microscopic examination of the sporulating cells after 30 hours revealed an even more pronounced cytological suppression with a large percentage of ∆*spoVFA* ∆*gerA* (or ∆*spoVFB* ∆*gerA*) spores appearing phase grey-white (Fig 1A and S1 Fig).

The *ylbJ* gene encodes a membrane protein of unknown function that is expressed under the control of the mother cell transcription factor SigE [25]. Based on the characterization of *ylbJ* described below we have renamed the gene *spoVV*. The ∆*spoVV* mutant is one of the last uncharacterized sporulation mutants that has a strong defect in spore formation. Cells lacking SpoVV are reduced in sporulation efficiency by >1,000,000-fold as assayed by resistance to wet heat [25, 26]. However, in the absence of the GerA receptor the sporulation efficiency of the ∆*spoVV* mutant improved dramatically (>15,000-fold) to 0.33% [24] (Fig 1A). As was the case for ∆*spoVFA*, a strong cytological suppression was also observed in the ∆*spoVV* ∆*gerA* double mutant (Fig 1A). Since ∆*gerA* suppressed both ∆*spoVFAB* and ∆*spoVV* and restored sporulation efficiency to similar levels, we wondered whether SpoVV could be involved in DPA accumulation in the forespore.

To investigate this possibility, we sought to quantitatively compare the DPA levels in spores derived from wild-type and the ∆*spoVV* and ∆*spoVFA* mutants. For these assays, concentrated spore suspensions with equivalent optical densities are boiled to release DPA, which is then quantified using a colorimetric assay [27, 28]. To prevent premature germination in the ∆*spoVFA* and ∆*spoVV* mutants we included a ∆*gerA* mutation. However, even in this background sporulated cells had many phase dark and hollow spores [24] (Fig 1A) making it difficult to normalize spore preparations using optical density. To circumvent this problem, we explored different sporulation methods and discovered that induction of sporulation by resuspension in which exponentially growing cultures are resuspended in defined minimal medium [29] yielded ~10-fold higher sporulation efficiencies and nearly homogenous populations of spores with a similar grey-white appearance (Fig 1B and S2 Fig).

Analysis of DPA content in these homogenous spore populations revealed that the ∆*spoVFA* mutant was reduced ~20-fold in DPA content (Fig 1C), as reported previously [30]. Furthermore, consistent with the idea that SpoVV is involved in DPA accumulation, spores lacking this protein had significantly reduced DPA levels (Fig 1C) although the levels were somewhat higher than a ∆*spoVFA* mutant. Importantly, two other mutants (∆*spoVT* and ∆*ytaF*) that were also suppressed by ∆*gerA* [24] produced spores with DPA levels similar to wild-type. These data indicate that SpoVV is required for accumulation of DPA in dormant spores.

### SpoVV localizes in the outer forespore membrane

**Fig 2:**
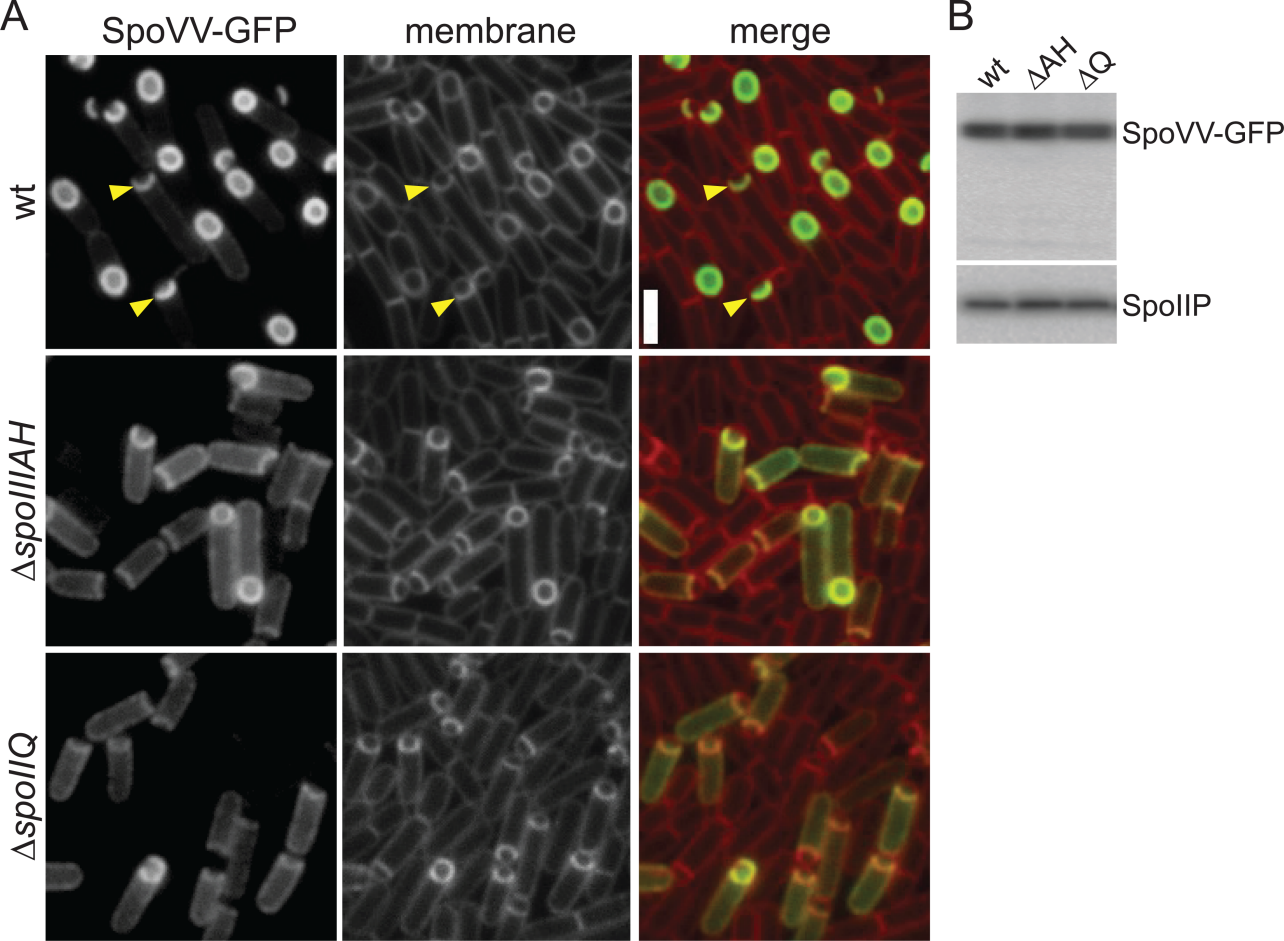
SpoVV-GFP localizes to the outer forespore membrane in a manner that depends on the SpoIIIAH and SpoIIQ. A. Representative images of SpoVV-GFP visualized by fluorescence microscopy at hour 3 of sporulation in wild-type (wt) (BDR3469), *∆spoIIIAH* (BDR3474), and *∆spoIIQ* (BDR3472) backgrounds. Examples of SpoVV-GFP localized in the engulfing membranes are highlighted (yellow carets). The membranes from the same fields were visualized with the fluorescent dye TMA-DPH (false-colored red). Scale bar indicates 2 μm. B. The SpoVV-GFP fusion remains full-length and at similar levels in all three strains. Immunoblot analysis of whole cell lysates using anti-GFP antibody from the indicated strains collected at hour 3 after the onset of sporulation. The SpoIIP protein, produced under the control of SigE, was used to control for loading.

SpoVV is a polytopic membrane protein with remote homology [31] to concentrative nucleoside transporters (see below). Based on the reduction of DPA levels in spores lacking SpoVV, we hypothesized that it could transport DPA across the mother cell membrane that surrounds the forespore. One prediction of this model is that SpoVV should specifically localize in the outer forespore membranes. To investigate this, we constructed a functional SpoVV-GFP fusion and analyzed its localization during a sporulation time-course. SpoVV-GFP localized to the septal membranes at the onset of engulfment and accumulated in the outer forespore membrane throughout this phagocytic-like process (Fig 2A).

This localization pattern has been reported for a large number of membrane proteins that are produced in the mother cell under the control of SigE [5, 32–36]. In several cases, a protein complex composed of SpoIIQ and SpoIIIAH is required to anchor these membrane proteins at this subcellular site [33, 34]. SpoIIQ is produced in the forespore and SpoIIIAH in the mother cell [37, 38]. These two bitopic membrane proteins interact across the intermembrane space and function as a localization hub for membrane proteins in the inner [39] and outer forespore membranes. Accordingly, we wondered whether SpoVV-GFP localization requires this trans-envelope complex. In support of this idea, the fusion protein was largely mislocalized in the absence of either SpoIIQ or SpoIIIAH (Fig 2A). Importantly, immunoblot analysis revealed that the SpoVV-GFP fusion protein remained intact in the two mutants (Fig 2B). Based on these findings, we propose that like other membrane proteins produced under SigE control, SpoVV is inserted into the peripheral membranes and then becomes localized to the outer forespore membrane by diffusion and capture by the SpoIIIAH-SpoIIQ complex [40].

### Localization of SpoVV to the peripheral membranes of the mother cell leads to secretion of DPA into the culture medium

**Fig 3:**
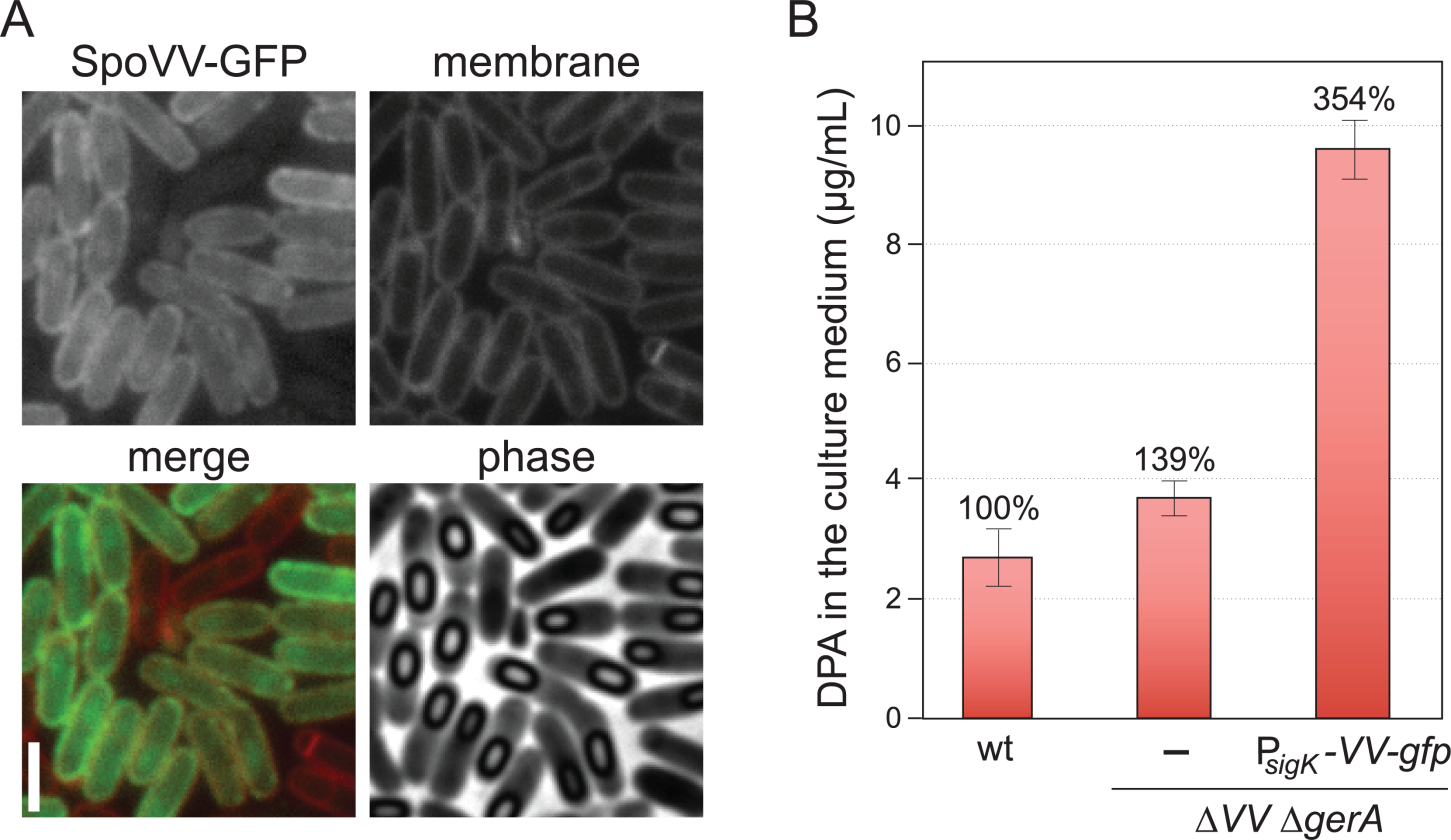
Localization of SpoVV-GFP to the cytoplasmic membranes of the mother cell during sporulation leads to DPA accumulation in the culture medium. A. SpoVV-GFP localization in a strain (BDR3471) in which the gene fusion was expressed under the control of the late-acting mother-cell sigma factor SigK. SigK becomes active after engulfment is complete and the outer forespore membranes and cytoplasmic membranes become topologically distinct. SpoVV-GFP was visualized by fluorescence microscopy at hour 7 of sporulation. The membranes from the same field were visualized with the fluorescent dye TMA-DPH (false-colored in red in the merge panel). Phase contrast images highlight phase-bright spores within the mother cell cytoplasm. Scale bar indicates 2 μm. B. Bar graph showing the amount of DPA in the culture supernatant of the indicated strains. Results shown are the average ± standard deviation of two independent biological replicates.

To investigate whether SpoVV is involved in membrane transport of DPA, we sought to localize the protein to the peripheral membranes of the mother cell. If SpoVV functions in DPA transport, DPA should accumulate in the sporulation medium under these conditions. To specifically localize SpoVV in the peripheral membranes, we took advantage of an observation made over a decade ago that membrane proteins produced in the mother cell after engulfment is complete are inserted exclusively into the peripheral membrane and are unable to re-localize around the outer forespore membrane because these membrane compartments are topologically distinct [40]. We fused *spoVV*-*gfp* to a promoter (P_*yeeK*_) recognized by SigK [2] that becomes active in the mother cell shortly after the completion of engulfment. Under these conditions, SpoVV-GFP was only detectable starting at hour 5 of sporulation in mother cells in which membrane fission had occurred and the engulfed forespore was released into the mother cell cytoplasm (Fig 3A). As anticipated, SpoVV-GFP was almost exclusively localized in the peripheral membranes of the sporangium (Fig 3A).

To investigate whether DPA accumulates in the sporulation medium, we harvested cultured supernatant from sporulating cells at hour 7 and analyzed DPA levels. In cells lacking SpoVV, there was a small increase in DPA in the culture medium compared to wild-type [41]. However, in cells expressing SpoVV-GFP the levels of secreted DPA were 3.5-fold higher than wild-type. These results are consistent with the idea that SpoVV or a protein that interacts with it transports DPA across the membrane.

### SpoVV-dependent secretion of DPA during vegetative growth

**Fig 4:**
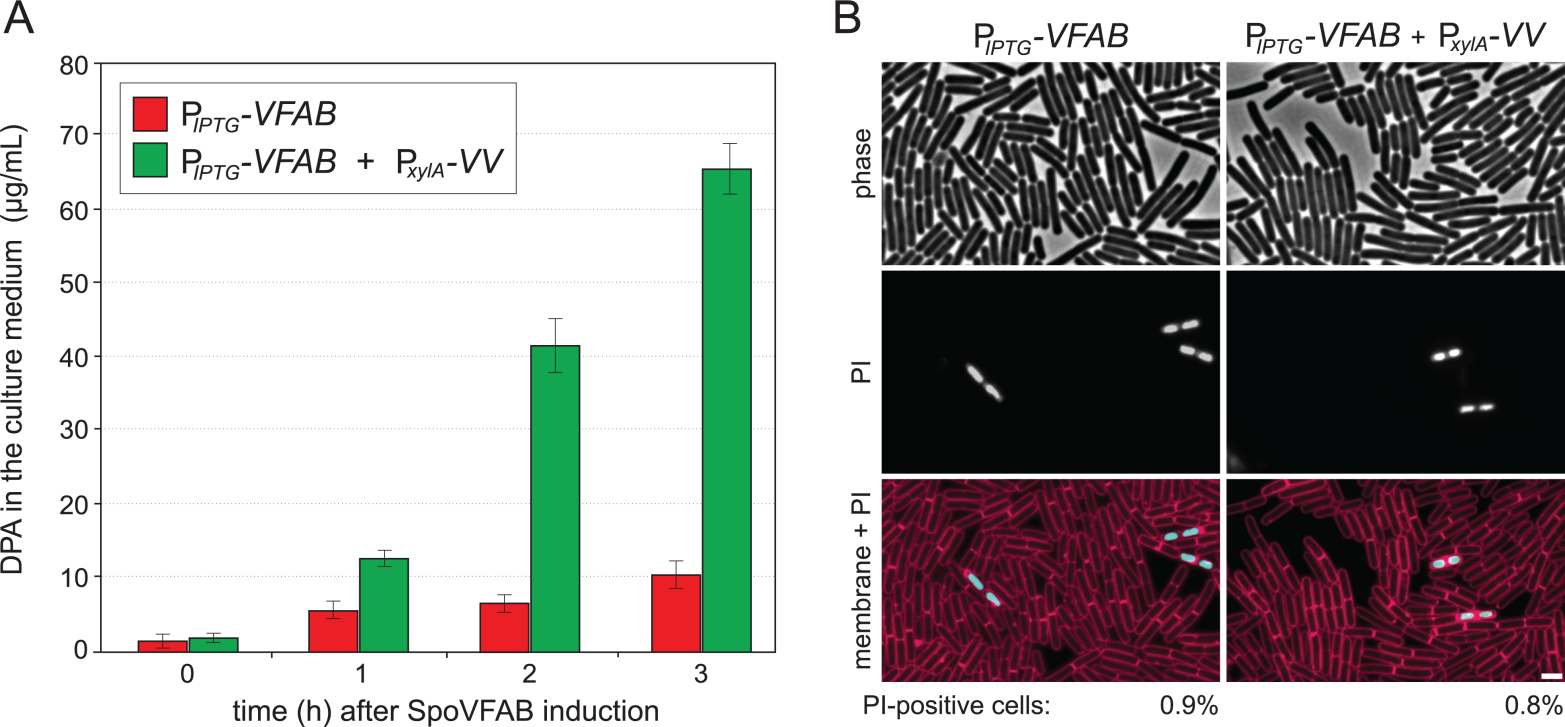
Expression of SpoVV and the SpoVFAB during vegetative results in secretion of DPA into the medium. A. Bar graph showing the amount of DPA in the culture supernatant before and every hour after the induction of SpoVFAB. Strains harboring a P_*hyperspank*_ fusion to *spoVFAB* (P_*IPTG*_-*VFAB*) encoding the DPA synthase with (BDR3432) or without (BDR3430) a P_*xylA*_ fusion to *spoVV* (P_*xylA*_-*VV*) were grown in minimal medium containing 33mM xylose. When the cultures reached an OD_600_ of 0.3, IPTG was added (0.5mM, final concentration) to induce expression of the DPA synthase. 2 mL of culture were removed every hour (before and after IPTG addition) and the supernatant was assayed for DPA content. Results shown are the average ± standard deviation of three independent biological replicates. B. Representative phase-contrast and fluorescence microscopy images of the indicated strains 3 h after the addition of IPTG. Membranes were stained with TMA-DPH (false-colored red) and membrane permeability was assessed with propidium iodine (PI) (false-colored blue). The frequencies of PI-positive cells are indicated below the images (*n* >1,500 cells for each strain). Growth curves and images of wild-type cells can be found in S3 Fig. Scale bar indicates 2 μm.

To investigate whether any other sporulation proteins are required for SpoVV-dependent transport of DPA, we sought to reconstitute DPA secretion in vegetatively growing cells. A previous study found that expression of SpoVFAB under non-sporulating conditions was sufficient to produce DPA [21]. Accordingly, we fused the *spoVFAB* operon to a strong IPTG-inducible promoter (P_*hyperspank*_) and *spoVV* to a xylose-inducible promoter (P_*xylA*_). Both fusions were inserted in single copy in the *B*. *subtilis* genome. Cells harboring P_*hyperspank*_-*spoVFAB* with and without P_*xylA*_*-spoVV* were grown in minimal medium in the presence of xylose. DPA accumulation in the culture medium was then analyzed before and every hour after the addition of IPTG and expression of the DPA synthase. In support of the idea that SpoVV is the only sporulation-specific protein required for DPA transport, DPA accumulated to high levels in the medium during the induction time course (Fig 4A). Production of DPA reduces the pools of lysine and *meso*-diaminopimelic acid (mDAP) required for cell wall synthesis [42]. Accordingly, to investigate whether SpoVFAB expression caused lysis and release of DPA, we examined the cells by fluorescence microscopy. No lysis was observed and cell morphology was largely similar to wild-type (Fig 4B and S3 Fig). A small percentage of cells had defects in membrane permeability as assayed by propidium iodide (PI). However, the percentages of PI-positive cells were similar between the two strains and to wild-type cells (Fig 4B and S3 Fig).

### SpoVV resembles nucleotide concentrative transporters

**Fig 5:**
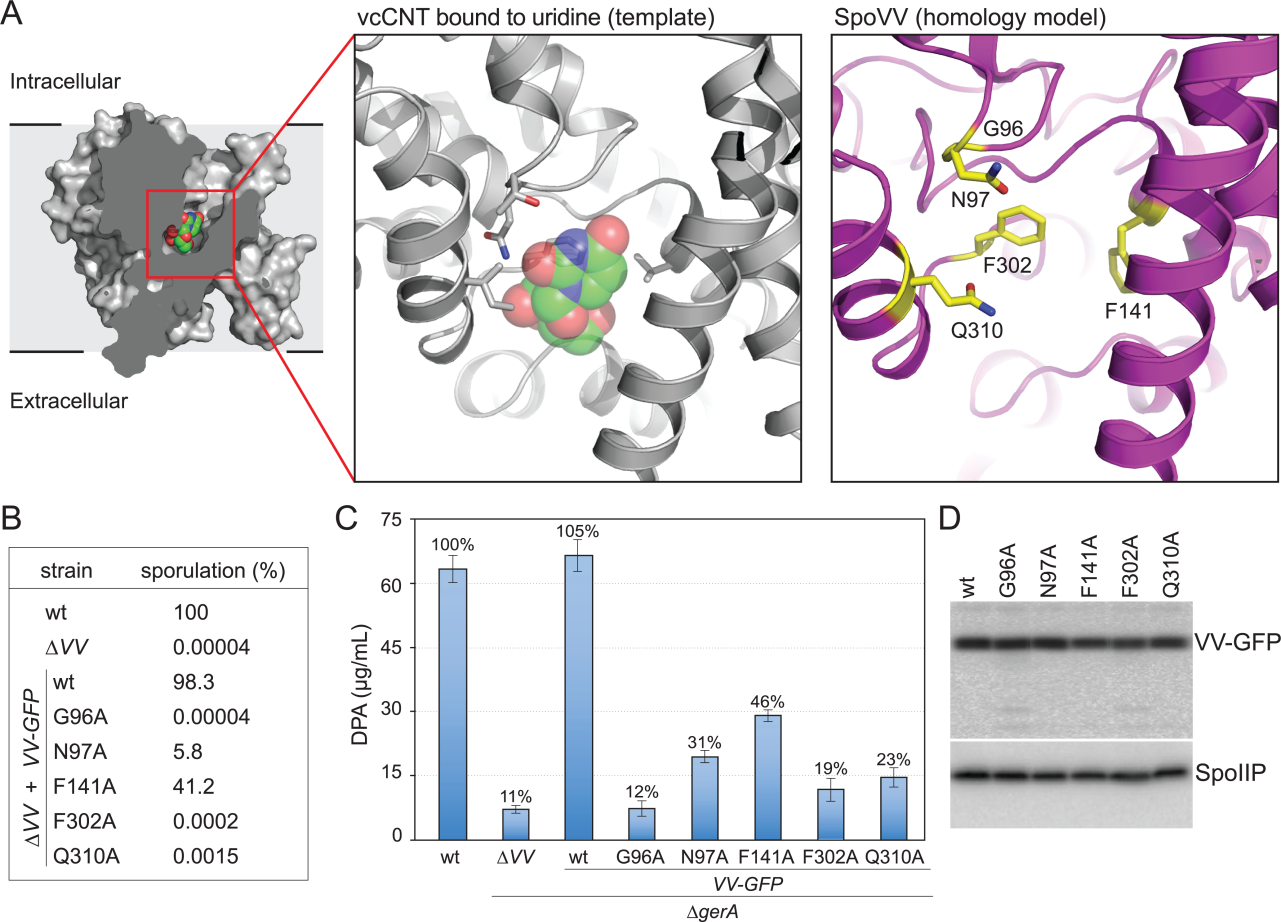
SpoVV resembles concentrative nucleoside transporters. A. Structure of the *V*. *cholerae* concentrative nucleoside transporter (vcCNT) in cross-section bound to uridine in the inward open orientation. Close-up of the substrate-binding pocket of vcCNT bound to uridine and the putative substrate-binding pocket of SpoVV highlighting amino acid residues (yellow) predicted to interact with DPA. B. Sporulation efficiencies of the indicated strains harboring amino acid substitutions in the putative substrate-binding pocket of SpoVV. The mutations were generated in the context of the *spoVV-gfp* fusion to enable the assessment of protein levels. Cells were sporulated in liquid medium at 37°C and sporulation efficiency (assessed by heat-resistance to 80°C for 20 min) was determined. C. Bar graph showing DPA content in dormant spores of the indicated strains. Purified spores were generated by resuspension and DPA was assayed as described in Fig 1. All *spoVV* mutant strains lacked the B subunit of the GerA receptor (designated ∆*gerA* for clarity) to prevent premature germination. Results shown are the average ± standard deviation of two independent biological replicates. D. Immunoblot showing the levels of the SpoVV-GFP mutants (in the ∆*spoVV* ∆*gerAB* background) compared to wild-type. Whole cell lysates from sporulating cells collected 3 h after the onset of sporulation were analyzed using anti-GFP antibodies. SpoIIP levels were used to control for loading. We note that as reported previously [75], small differences in DPA levels appear to have dramatic effects on heat resistance (compare, for example, SpoVV(N97A) and SpoVV(Q310A)). The defects in sporulation efficiencies reported here and likely those reported previously reflect both the loss in heat resistance and the percentage of sporulating cells in which the GerA receptor was prematurely activated [24]. Analysis of sporulation efficiency of the SpoVV mutants in a strain lacking GerAB followed similar trends.

SpoVV is predicted to contain eight transmembrane segments and is annotated as a member of the Gate family of transporters (PFAM07670). Remote homology searches using HHPred [31] identified high confidence hits (99.67% probability, E-value 7.5e-16) to concentrative nucleoside transporters in the Gate family. These polytopic membrane proteins are found in all domains of life and couple ion transport to the uptake of nucleosides against their concentration gradients [43, 44]. Characterized family members include the human nucleoside transporters hCNT1 and hCNT2 that transport nucleosides and mediate uptake of nucleoside-derived drugs [45–47] and the concentrative nucleoside transporters vcCNT and CNT_NW_ from *Vibrio cholerae* and *Neisseria wadsworthii*, respectively, for which high resolution crystal structures have been determined [48–50]. These so-called elevator-type transporters are thought to move their substrates across the membrane by alternating access of their binding sites between the two sides of the membrane. This is accompanied by a large movement of the substrate-binding transport domain from one side of the bilayer to the other. To further investigate the idea that SpoVV is the DPA transporter, we used the structure of vcCNT bound to uridine [50] to model SpoVV (Fig 5A). The threaded SpoVV structure identified five residues predicted to be located in the substrate-binding site (Fig 5A). To determine whether these amino acids are important for function, we generated site-directed mutations in the context of the *spoVV-gfp* fusion and tested whether they could complement the ∆*spoVV* null mutant. Three of the five SpoVV mutants (G96A, F302A, and Q310A) had strong sporulation defects while the other two (N97A, and F141A) had more mild phenotypes (Fig 5B). Analysis of DPA content in spores derived from these mutants (and lacking the GerA receptor) revealed reduced levels of DPA (Fig 5C). Importantly, all five mutant proteins were produced at levels similar to wild-type (Fig 5D). Moreover, the extent of reduction in DPA levels in the mutants followed the same trend as the reduction in sporulation efficiency. Collectively, these data support the idea the SpoVV binds DPA and likely functions to transport it across the outer forespore membrane.

## Discussion

Since its discovery in 1953, DPA has been found to be both a universal and specific component of bacterial endospores [7, 51, 52]. For over forty years it has been appreciated that this unusual molecule is produced in the mother cell at a late stage of sporulation [53] but its intracellular transport into the developing spore has remained unclear. Here, we provide evidence for a simple two-step transport pathway in which SpoVV translocates DPA across the outer forespore membrane followed by its import into the forespore by the proteins encoded by the *spoVA* locus. While it is possible that SpoVV and the SpoVA proteins interact across the intermembrane space, we favor the idea that they function independently. During engulfment, the distance between the inner and outer membranes is already quite large (~22 nm) [54] and cortex assembly, which occurs at the same time as DPA transport, would further increase the distance between these two membranes. Thus, we envision DPA transiently accumulates in the intermembrane space prior to its uptake into the forespore. This two-step transport pathway is likely to be a broadly conserved mechanism by which the mother cell nurtures the forespore as virtually all endospore formers have a SpoVV ortholog and a core set of the SpoVA proteins: SpoVAC, SpoVAD, SpoVAEb and SpoVAF (S4 Fig). Interestingly, most but not all endospore formers have SpoVFA and SpoVFB orthologs. However, those that lack these proteins appear to use a distinct enzyme (EtfA) to synthesize DPA [55].

### SpoVV-SpoVA transport and the SpoIIIA-SpoIIQ complex

We contrast the simple intracellular transport pathway described here with a seemingly more complicated one that has been proposed to function at an earlier stage in sporulation. During engulfment a complex composed of SpoIIQ synthesized in the forespore and the proteins in the SpoIIIA operon and GerM produced in the mother cell is assembled across the inner and outer forespore membranes [5, 33, 34, 56]. Several proteins in this trans-envelope complex resemble components of specialized secretion systems found in Gram-negative bacteria [5, 6, 57] and three of them SpoIIQ, SpoIIIAH, and SpoIIIAG appear to form a channel in the space between the inner and outer forespore membranes [58–61]. It has thus been proposed that this complex functions as a feeding tube or secretion system to provide mother-cell-produced molecules to the forespore [4–6]. In support of this idea, the complex is required to maintain forespore gene expression and in its absence the forespore fails to thrive, never reaches its full size, and develops membrane invaginations [4, 5, 62]. To date, the molecules transported by this complex remain unknown. Like the DPA transport pathway, the SpoIIIA proteins are among the most broadly conserved sporulation factors [63]. Our findings lead us to wonder why endospore-formers have evolved and retained such a complex machine to transport molecules if a simpler one could have replaced it? One possibility is that this complex transports mother-cell produced proteins rather than small molecules like DPA. Alternatively, it has recently been suggested [60] that this complex might not be involved in secretion but rather function as a specialized piliation system to physically hold the inner and outer forespore membranes together. Defining the role of this broadly conserved and enigmatic complex and whether or not it transports mother-cell-produced molecules awaits further investigation.

### DPA transport highlights the logic of the sporulation pathway

**Fig 6:**
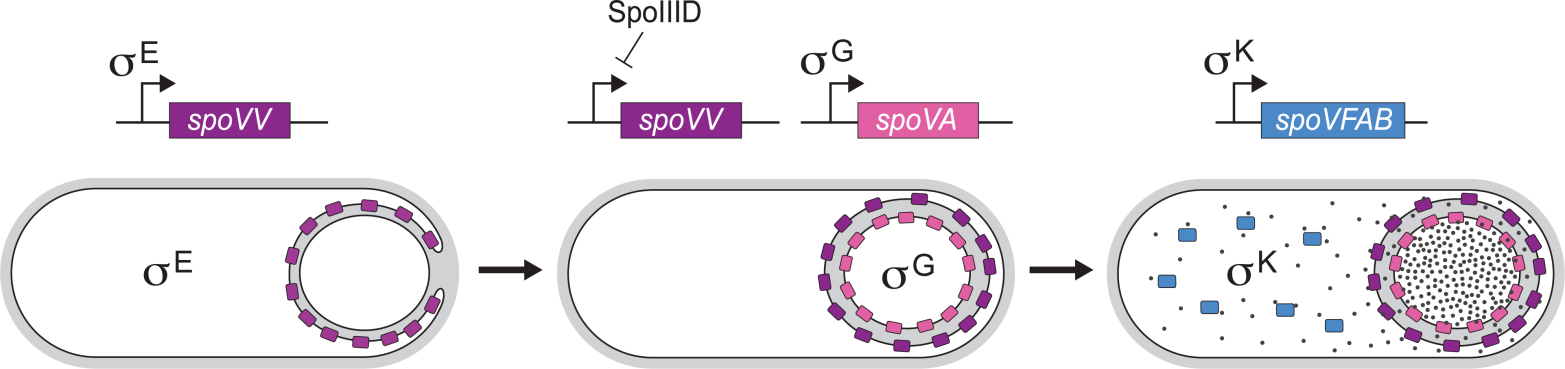
The DPA transport pathway during spore morphogenesis. Schematic model of the spatio-temporal regulation of the DPA transport pathway and forespore accumulation of DPA. During the morphological process of engulfment, SpoVV is produced in the mother cell under SigE control where it specifically localizes in the outer forespore membrane in a manner that depends on the SpoIIQ-SpoIIIAH complex (not shown) (left panel). Prior to the completion of engulfment, SpoIIID represses transcription of the *spoVV* gene. Accordingly, upon completion of engulfment SpoVV is no longer produced and cannot accumulate in the cytoplasmic membranes of the mother cell. After completion of engulfment, the proteins of the *spoVA* operon are produced in the forespore under the control of SigG (middle panel). Finally, SpoVFAB proteins are produced in the mother cell under the control of SigK leading to the synthesis of DPA (dark circles). DPA is then transported across the two membranes that separate the mother cell and forespore.

The DPA transport pathway described here exemplifies how intimately the sporulation gene expression programs are coordinated with morphogenesis (Fig 6). The SpoVV transporter is produced early during sporulation under the control of the mother cell sigma factor SigE [25]. Synthesis at this time ensures that the protein achieves its proper subcellular localization, presumably by diffusion and capture in the outer forespore membranes (Figs 2 and 3) [40]. SigE also activates a mother cell transcription factor SpoIIID that functions as both a positive and negative regulator of mother cell gene expression [64]. Transcriptional profiling indicates that most genes encoding membrane proteins in the SigE regulon, including *spoVV*, are repressed by SpoIIID shortly after they are activated [19]. Thus, SpoVV production is shut off prior to the completion of engulfment ensuring that most if not all of the proteins are positioned to transport DPA into the intermembrane space rather than secrete it into the medium (Fig 6) [40]. After membrane fission, SigG becomes active and is responsible for the synthesis of the SpoVA proteins in the forespore [65]. And finally, SpoVFA and SpoVFB are synthesized in the mother cell [21]. Production of the DPA synthase at this late stage in spore development has two consequences. First, it guarantees that the DPA transported into the intermembrane space will not leak into the medium, as would be the case prior to the completion of engulfment. Second, since DPA synthesis drains intermediates from the lysine and mDAP biosynthetic pathways, it ensures that the production of high levels of DPA occurs when most mother cell gene expression is near complete and the mother cell is soon to lyse. Thus, accumulation of DPA in the developing spore involves three enzymes that are produced under the control of three distinct sporulation sigma factors at different stages in development. This spatio-temporal regulation ensures efficient DPA transport into the forespore and ultimately the production of dormant, stable, and highly resistant spores.

## Methods

### General methods

*B*. *subtilis* strains were derived from the auxtrophic strain168 [66]. Sporulation was induced in liquid medium at 37°C by nutrient exhaustion in supplemented DS medium (DSM) [67] or by resuspension according to the method of Sterlini-Mandelstam [29]. Sporulation efficiency was determined in 24–30 h cultures as the total number of heat-resistant (80°C for 20 min) colony forming units (CFUs) compared to wild-type heat-resistant CFUs. Insertion-deletion mutants were from the *Bacillus* knock-out (BKE) collection [68] or were generated by isothermal assembly [69] of PCR products followed by direct transformation into *B*. *subtilis*. All BKE mutants were back-crossed twice into *B*. *subtilis* 168 before assaying and prior to antibiotic cassette removal. Antibiotic cassette removal was performed using a temperature-sensitive plasmid that constitutively expresses Cre recombinase [70]. Unless otherwise indicated, *B*. *subtilis* strains were constructed using genomic DNA and a 1-step competence method. Tables of strains (S1 Table), plasmids (S2 Table) and oligonucleotide primers (S3 Table) can be found in Supplemental Information. A description of plasmid and strain constructions is provided in S1 Methods.

### Microscopy

Vegetative and sporulating cells and dormant spores were collected by centrifugation at 6,500×g for 1 min and immobilized on 2% agarose pads. Phase-contrast and fluorescence microscopy were performed using an Olympus BX61 microscope equipped with an UplanF1 100× phase contrast objective lens and a monochrome CoolSnapHQ digital camera (Photometrics). Membranes were stained with 1-(4-trimethylammoniumphenyl)-6-phenyl-1,3,5-hexatriene *p*-toluenesulfonate (TMA-DPH; Molecular Probes) at a final concentration of 50μM. Live-dead staining was performed with propidium iodide (PI; Invitrogen) at a final concentration of 5μM. Exposure times were typically 100, 200 and 400 ms for TMA-DPH, GFP, and PI, respectively. Image analysis and processing were performed using MetaMorph software (Molecular Devices; version 7.7).

### Spore purification

Spores produced by resuspension at 37°C were harvested 30 h after the onset of sporulation. The spore pellet was suspended in 25 mL of TE containing 30 mg of lysozyme, incubated for 1 h at 37°C with shaking and then 3 mL of 20% SDS was added and incubated for an additional 20 min. The spore suspension was exhaustively washed with ddH_2_O prior to assaying and/or imaging.

### Quantification of DPA

Spore DPA content was assayed as described previously [28]. Briefly, 1 mL of spores at an OD_600_ of 5 was incubated at 100°C for 30 min and then placed on ice for 15 min. The spores were pelleted by centrifugation (16,000 ×g for 10 min) and the supernatant was assayed for DPA content. For the experiments in which DPA was assayed in the culture medium, 2 mL of vegetatively growing or sporulating cells were pelleted by centrifugation (16,000×g for 10 min) and the supernatant was assayed directly for DPA content. The supernatant (400 μL) was mixed with an equal volume of ddH_2_O and then incubated with 200 μL of solution 1 containing 50 mM sodium acetate (pH 4.6), 8.25 mM L-cysteine, 24.22 mM (NH_4_)_2_SO_4_ and 44.76 mM FeSO_4_. The reaction was incubated at room temperature for 2 min and the supernatant was then collected by centrifugation (16,000×g for 5 min) and its absorbance measured at 440 nm. Commercial DPA (Sigma-Aldrich) was used to generate a standard curve. Because rich medium interferes with the colorimetric assay, for the experiments in which DPA levels were assayed in the culture supernatant, minimal medium was used for the vegetatively growing cells and sporulation was induced by resuspension.

### Homology modeling

To generate a homology model of SpoVV, the sequence was first aligned to that of *Vibrio cholerae* concentrative nucleoside transporter. The structure of the transporter in complex with uridine (PDB ID 4PD6; [50]) was used as a template for homology modeling using the SWISS-MODEL server [71]. The template structure and homology model were aligned, and non-alanine residues within a 5 Å radius of uridine in the template structure were selected as candidates for mutagenesis.

### Immunoblots assays

Sporulating cells induced by resuspension were collected at hour 3 of sporulation by centrifugation and whole-cell lysates were prepared as described previously [72]. Samples were heated for 10 min at 55°C prior to loading. Equivalent loading was based on OD_600_ at the time of harvest. Proteins were separated by SDS-PAGE on 12.5% polyacrylamide gels, electroblotted onto Immobilon-P membranes (MilliporeSigma) and blocked in 5% non-fat milk in phosphate-buffered saline (PBS)-0.5% Tween-20. The blocked membranes were probed with anti-GFP [73] and anti-SpoIIP [74] antibodies diluted 1:10,000 into 3% BSA in 1× PBS-0.05% Tween-20. Primary antibodies were detected using horseradish peroxidase-conjugated goat, anti-rabbit IgG (Bio-Rad) and the Western Lightning reagent kit as described by the manufacturer (PerkinElmer).

## Supporting information

S1 Fig Suppression of ∆*spoVFA* and ∆*spoVFB* in the absence of a functional GerA receptor. Representative phase-contrast images of the indicated strains sporulated for 30 h at 37°C in liquid DSM are shown. Sporulation efficiencies are indicated above each image. Strains lacking the B subunit of the GerA receptor (GerAB) are designated ∆*gerA* for clarity. Scale bar indicates 2 μm.
(PDF)

S2 Fig Sporulation by resuspension: Suppression of ∆*spoVV* and ∆*spoVFA* in the absence of a functional GerA receptor. Representative phase-contrast images of the indicated strains sporulated by resuspension for 30 h at 37°C. Sporulation efficiencies are indicated above each image. Strains lacking the B subunit of the GerA receptor (GerAB) are designated ∆*gerA* for clarity. We note that the homogenous spore populations shown in Fig 1B were achieved after spore purification (see Methods). Scale bar indicates 2 μm.
(PDF)

S3 Fig Expression of SpoVV and the SpoVFAB synthase during vegetative results in secretion of DPA into the medium. **A.** Growth curve of wild-type and the strains engineered to over-express the DPA synthase in the presence and absence of SpoVV. Indicated strains were grown in minimal medium supplemented with 33 mM xylose. When the cultures reached an OD_600_ of 0.3, IPTG was added (0.5 mM, final concentration) to induce expression of SpoVFA and SpoVFB. Samples were collected before and after IPTG addition at the indicated times to assay DPA levels in the medium. **B.** Representative phase-contrast and fluorescence microscopy images of the indicated strains collected 3 h after the addition of IPTG. Membranes were stained with TMA-DPH (false-colored red) and membrane permeability was assessed with propidium iodide (PI) (false-colored blue). The number of PI-positive cells was quantified for each strain. Scale bar indicates 2 μm.
(PDF)

S4 Fig Conservation of the DPA transport pathway in endospore-forming bacteria. Phylogenetic tree showing the co-occurrence of the DPA synthase (SpoVFA and SpoVFB) and transporters (SpoVV and SpoVAA-AF) in a diverse set of 1,773 bacterial taxa. The amino acid sequences of *B*. *subtilis* SpoVFA, SpoVFB, SpoVV, and the SpoVA proteins served as queries in a BLASTp search against the NCBI ‘nr’ database with an *e*-value cutoff of 1x10^-4^. This analysis was performed through the Harvard Medical School Research Computing Orchestra cluster. The phylogenetic tree was constructed in PhyloT (http://phylot.biobyte.de/) and the BLASTp search results were plotted against the tree. The tree was visualized and annotated using the Interactive Tree Of Life web-based tool (iTOL, v3; http://itol.embl.de).
(PDF)

S1 Methods Details for plasmid and strain constructions in this study. (PDF)

S1 Table Strains used in this study. (PDF)

S2 Table Plasmids used in this study. (PDF)

S3 Table Oligonucleotide primers used in this study. (PDF)
