# Supplementary figures and images for: A two-step transport pathway allows the mother cell to nurture the developing spore in Bacillus subtilis
Source: PLoS Genet. 2017 Sep 25;13(9):e1007015. doi: 10.1371/journal.pgen.1007015 (PMC5629000; doi:10.1371/journal.pgen.1007015)

wt (100%)

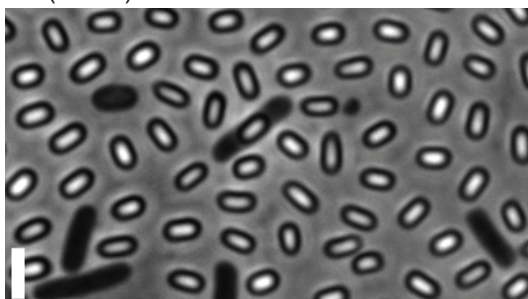

$\Delta gerA$  (86.1%)

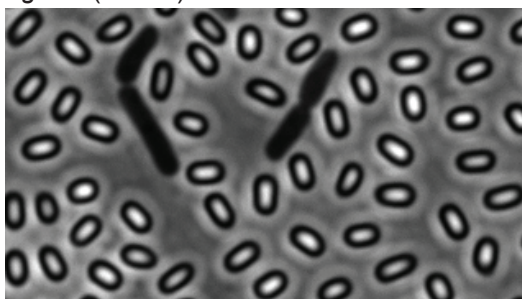

$\Delta spoVFA$  (0.0017%)

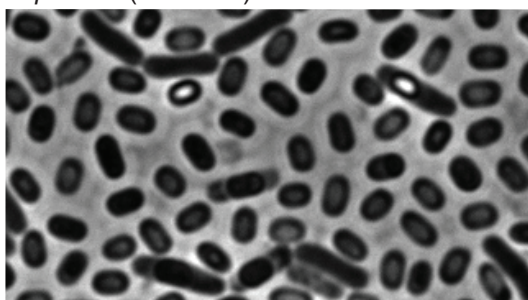

$\Delta spoVFA \Delta gerA$  (0.207%)

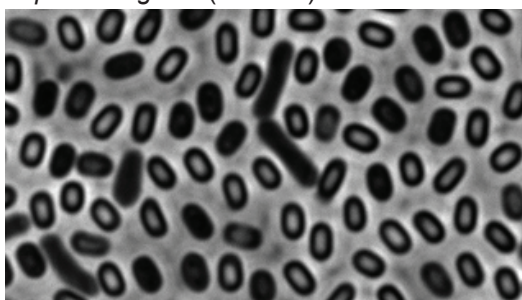

$\Delta spoVFB$  (0.0012%)

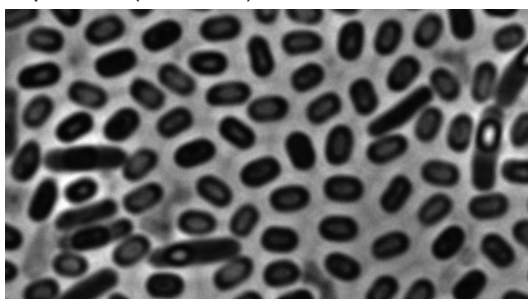

$\Delta spoVFB \Delta gerA$  (0.196%)

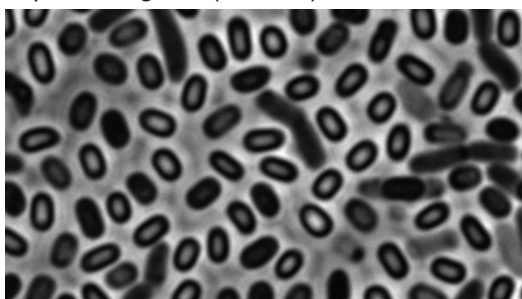

Supplement: S1 Fig — Representative phase-contrast images of the indicated strains sporulated for 30 h at 37°C in liquid DSM are shown. Sporulation efficiencies are indicated above each image. Strains lacking the B subunit of the GerA receptor (GerAB) are designated ∆gerA for clarity. Scale bar indicates 2 μm. (PDF) [file pgen.1007015.s001.pdf]

wt (100%)

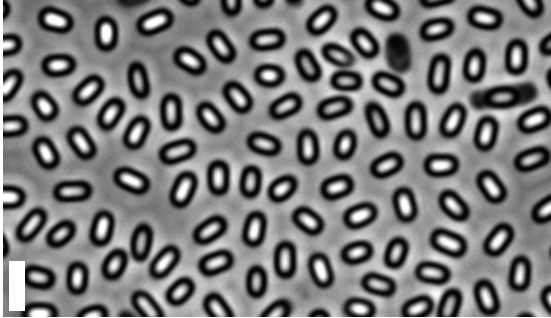

$\Delta gerA$  (13.9%)

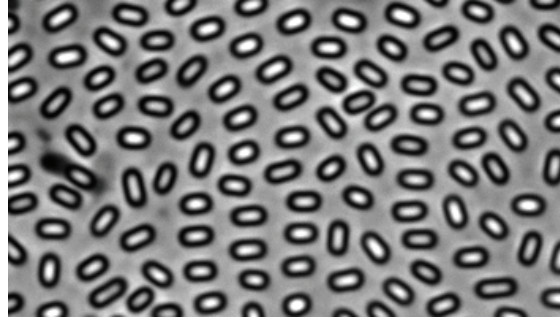

$\Delta spoVV$  (0.00008%)

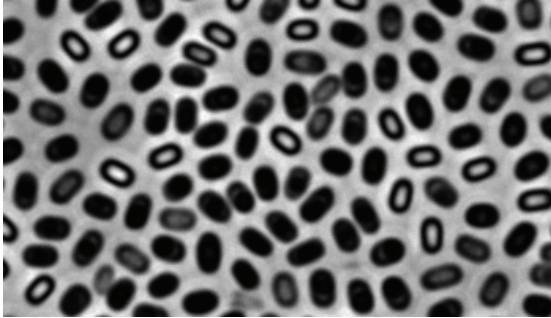

$\Delta spoVV \Delta gerA$  (2.96%)

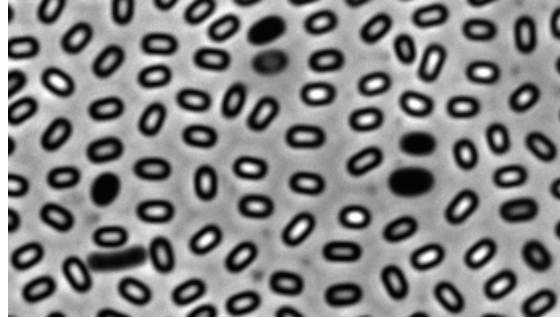

$\Delta spoVFA$  (0.004%)

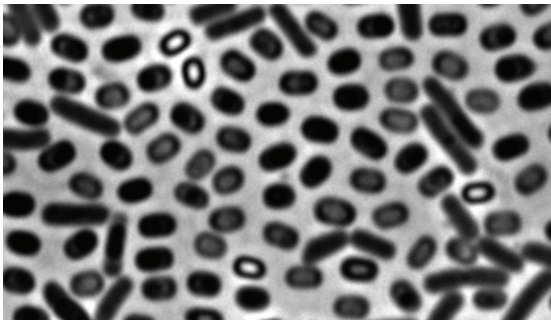

$\Delta spoVFA \Delta gerA$  (3.83%)

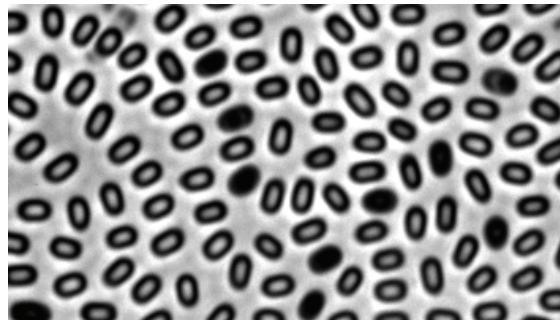

Supplement: S2 Fig — Representative phase-contrast images of the indicated strains sporulated by resuspension for 30 h at 37°C. Sporulation efficiencies are indicated above each image. Strains lacking the B subunit of the GerA receptor (GerAB) are designated ∆gerA for clarity. We note that the homogenous spore populations shown in Fig 1B were achieved after spore purification (see Methods). Scale bar indicates 2 μm. (PDF) [file pgen.1007015.s002.pdf]

A

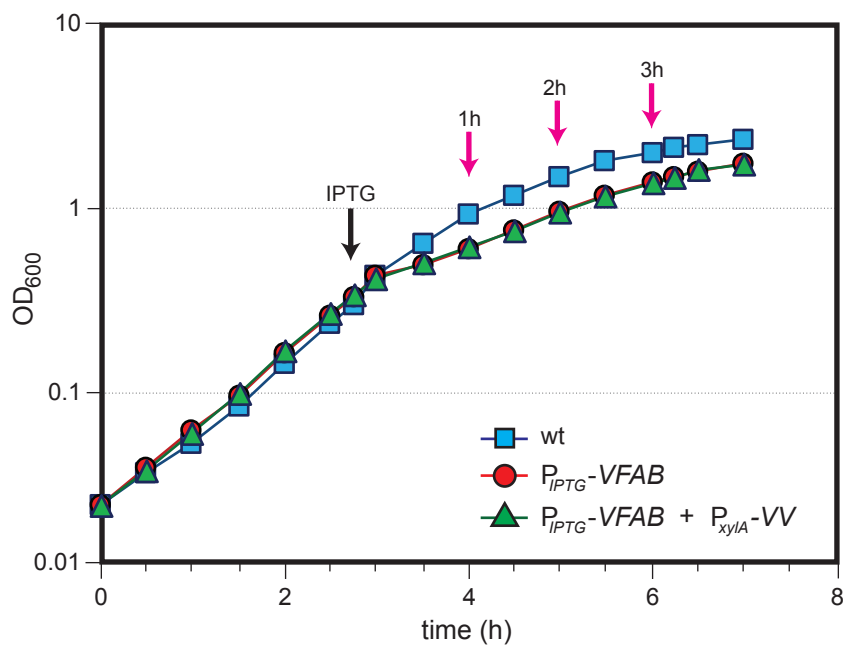

B

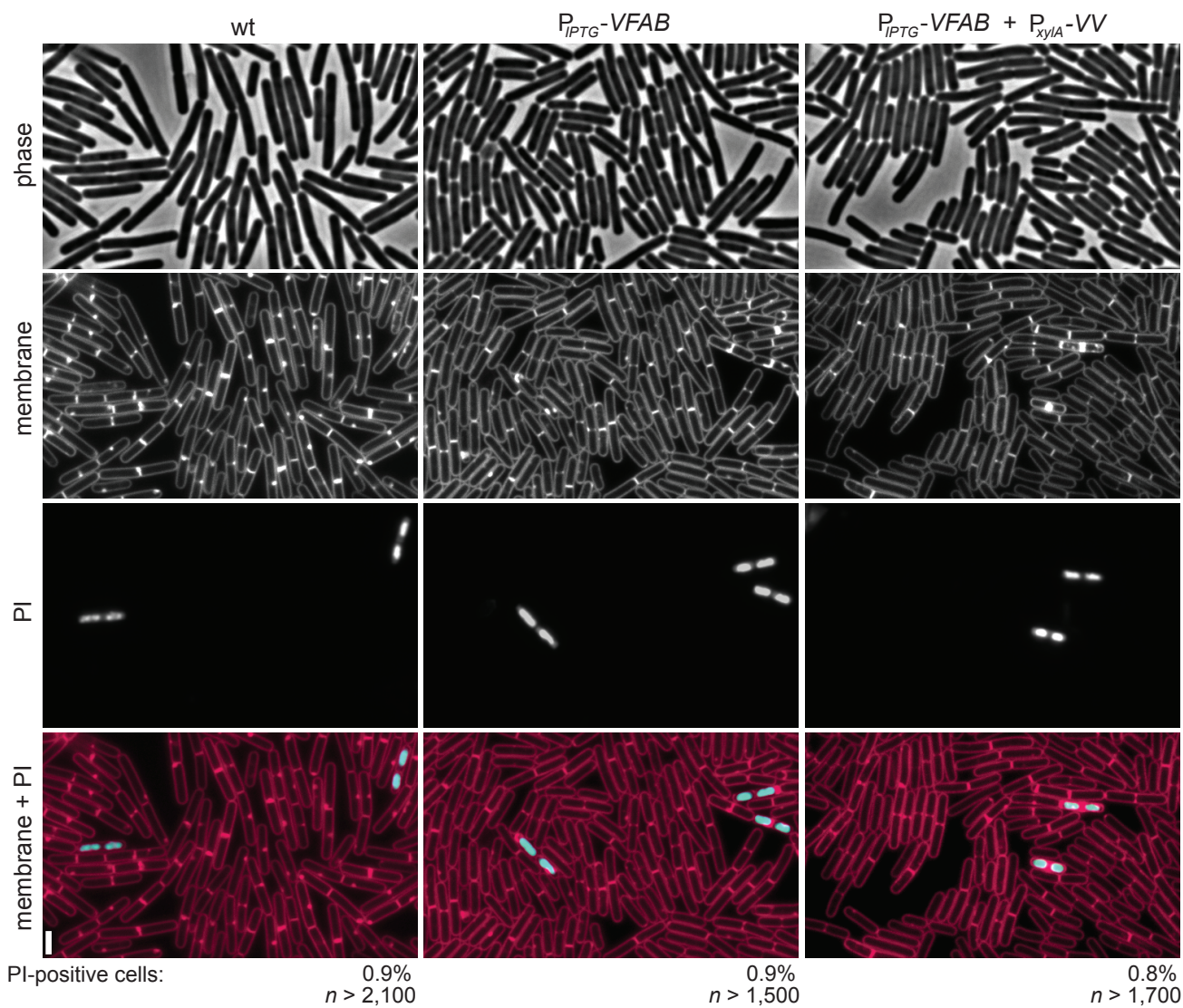

Supplement: S3 Fig — A. Growth curve of wild-type and the strains engineered to over-express the DPA synthase in the presence and absence of SpoVV. Indicated strains were grown in minimal medium supplemented with 33 mM xylose. When the cultures reached an OD600 of 0.3, IPTG was added (0.5 mM, final concentration) to induce expression of SpoVFA and SpoVFB. Samples were collected before and after IPTG addition at the indicated times to assay DPA levels in the medium. B. Representative phase-contrast and fluorescence microscopy images of the indicated strains collected 3 h after the addition of IPTG. Membranes were stained with TMA-DPH (false-colored red) and membrane permeability was assessed with propidium iodide (PI) (false-colored blue). The number of PI-positive cells was quantified for each strain. Scale bar indicates 2 μm. (PDF) [file pgen.1007015.s003.pdf]

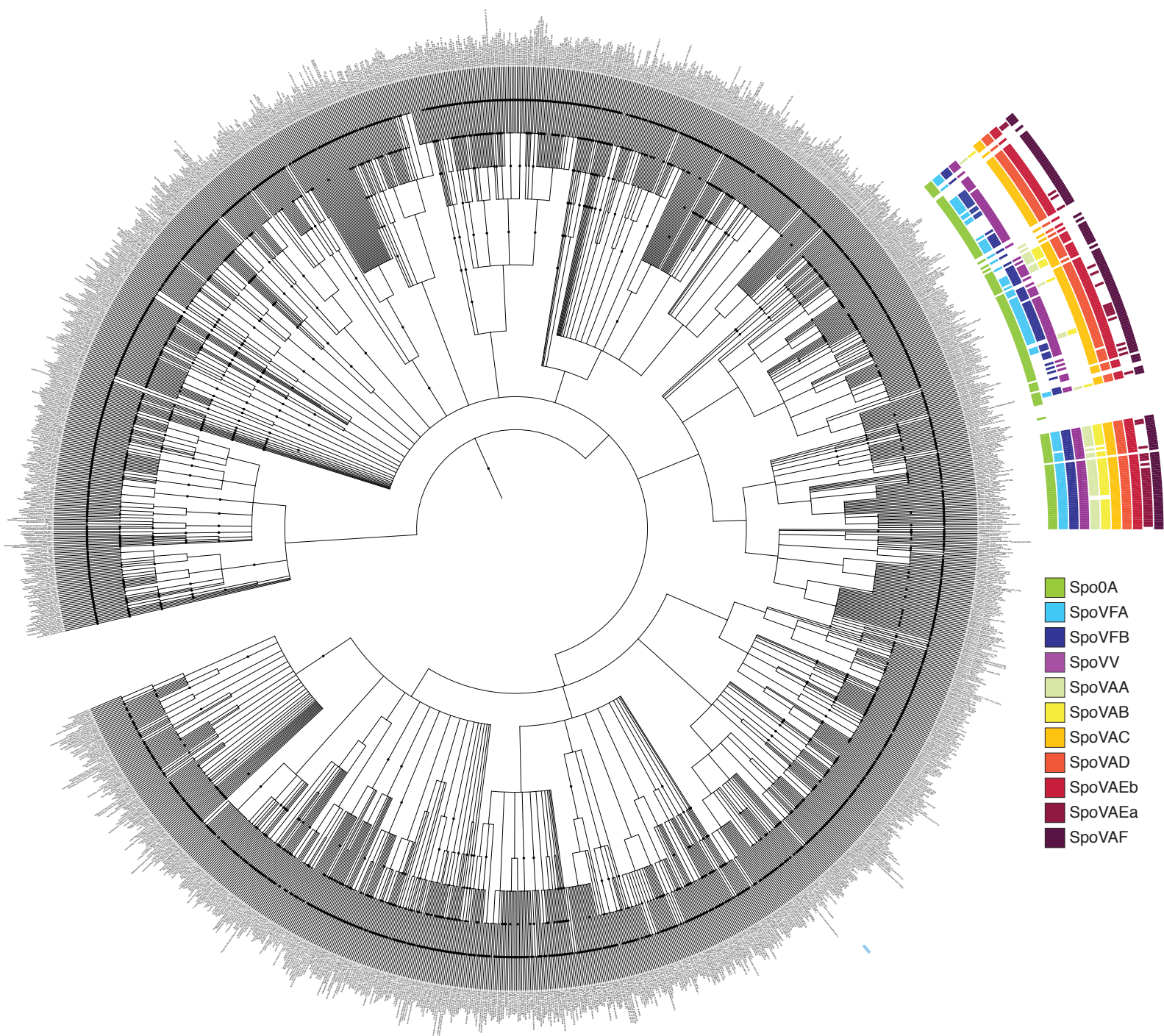

Supplement: S4 Fig — Phylogenetic tree showing the co-occurrence of the DPA synthase (SpoVFA and SpoVFB) and transporters (SpoVV and SpoVAA-AF) in a diverse set of 1,773 bacterial taxa. The amino acid sequences of B. subtilis SpoVFA, SpoVFB, SpoVV, and the SpoVA proteins served as queries in a BLASTp search against the NCBI ‘nr’ database with an e-value cutoff of 1x10^-4^. This analysis was performed through the Harvard Medical School Research Computing Orchestra cluster. The phylogenetic tree was constructed in PhyloT (http://phylot.biobyte.de/) and the BLASTp search results were plotted against the tree. The tree was visualized and annotated using the Interactive Tree Of Life web-based tool (iTOL, v3; http://itol.embl.de). (PDF) [file pgen.1007015.s004.pdf]
