# Supplementary material for: A two-step transport pathway allows the mother cell to nurture the developing spore in Bacillus subtilis
Source: PLoS Genet. 2017 Sep 25;13(9):e1007015. doi: 10.1371/journal.pgen.1007015 (PMC5629000; doi:10.1371/journal.pgen.1007015)
Supplement: S1 Methods — (PDF) [file pgen.1007015.s005.pdf]

## S1 Methods. Strains and plasmids construction

### Strains construction

Deletion mutants from the *Bacillus subtilis* knock-out collection were all confirmed by PCR using an oligonucleotide primer (oKO0) within the erythromycin resistance gene and a gene-specific primer.

**BDR3414** [Δ*spoVV* Δ*gerA::spec*] was generated by transforming *B. subtilis* BDR3154 (Δ*spoVV*) with a PCR product containing the *gerA::spec* mutation (amplified with oligonucleotide primers oFR5 and oFR8 and template DNA from BDR3371).

**BDR3416** [*ycgO::cat*] was generated by transforming *B. subtilis* 168 with pKM77. pKM77 (*ycgO::cat*) is a double-crossover vector for ectopic integration into the nonessential *ycgO* locus (Rudner Lab stock).

**BDR3430** [*ycgO::*$P_{hyperspank}$-*spoVFAB* (*erm*)] was generated by transforming *B. subtilis* BDR3416 with pFR001.

**BDR3432** [*ycgO::*$P_{hyperspank}$-*spoVFAB* (*erm*) *amyE*::$P_{xylA}$-*spoVV* (*spec*)] was generated by transforming *B. subtilis* BDR342 with pFR002.

**BDR3449** [*spoVV-gfp* (*spec*)] was generated by direct transformation of *B. subtilis* 168 with an isothermal assembly product derived from 3 PCR products: 1) a PCR product containing the *spoVV* gene lacking its stop codon amplified with oligonucleotide primers oDR1262 and oDR1263 and *B. subtilis* 168 genomic DNA as template; 2) a PCR product containing *gfp* (*mgfpmut3a*) and the *spec* cassette amplified with oligonucleotide primers oDR1264 and oDR1265 and DNA from pWX429a as template; 3) a PCR product containing the region downstream of *spoVV* amplified with oligonucleotide primers oDR1266 and oDR1267 and *B. subtilis* 168 genomic DNA as template.

**BDR3458** [Δ*spoIIQ::kan*] was generated by two back-crosses into *B. subtilis* 168 with genomic DNA from BCR267 (Rodrigues *et al.,* 2016)

**BDR3465** [*ycgO::*$P_{yeeK}$-*optRBS-spoVV-gfp* (*erm*)] was generated by transforming *B. subtilis* BDR3416 with pFR008.

**BDR3466** [*ycgO::*$P_{spoVV}$-*optRBS-spoVV-gfp* (*erm*)] was generated by transforming *B. subtilis* BDR3416 with pFR009.

**BDR3468** [Δ*spoVV ycgO::*$P_{yeeK}$-*optRBS-spoVV-gfp* (*erm*)] was generated by transforming *B. subtilis* BDR3154 with gDNA from BDR3465.

**BDR3469** [Δ*spoVV ycgO::*$P_{spoVV}$-*optRBS-spoVV-gfp* (*erm*)] was generated by transforming *B. subtilis* BDR3154 with gDNA from BDR3466.

**BDR3471** [Δ*gerA::spec* Δ*spoVV ycgO::*$P_{yeeK}$-*optRBS-spoVV-gfp* (*erm*)] was generated by transforming *B. subtilis* BDR3414 with gDNA from BDR3465.

**BDR3472** [Δ*spoVV* Δ*spoIIQ::kan ycgO::*$P_{spoVV}$-*optRBS-spoVV-gfp* (*erm*)] was generated by transforming *B. subtilis* BDR3469 with gDNA from BDR3458.

**BDR3474** [Δ*spoIIIAH* Δ*spoVV::spec ycgO::*P$_{spoVV}$-*optRBS-spoVV-gfp* (*erm*)] was generated in two steps: first, *B. subtilis* BCR1117 was transformed with gDNA from BDR3466, and this intermediate strain was transformed with a PCR product containing Δ*spoVV::spec* (amplified with oligonucleotide primers oKO260 and oFR3 and gDNA from BDR3312).

**BDR3507** [*ycgO::spoVV-gfp* (*spec*)] was generated by transforming *B. subtilis* BDR3416 with pFR11.

**BDR3527** [Δ*spoVV ycgO::spoVV-gfp* (*spec)*] was generated by transforming *B. subtilis* BDR3154 with gDNA from *B. subtilis* BDR3507.

**BDR3558** [Δ*spoVV ycgO::cat*] was generated by transforming *B. subtilis* BDR3154 with pKM77.

**BDR3562** [Δ*spoVV ycgO::spoVV*(*N97A*)-*gfp* (*spec*)] was generated by transforming *B. subtilis* BDR3558 with pFR017.

**BDR3563** [Δ*spoVV ycgO::spoVV*(*F302A*)-*gfp* (*spec*)] was generated by transforming *B. subtilis* BDR3558 with pFR018.

**BDR3564** [Δ*spoVV ycgO::spoVV-gfp* (*Q310A*) (*spec*)] was generated by transforming *B. subtilis* BDR3558 with pFR019.

**BDR3615** [Δ*spoVV ycgO::spoVV*(*F141A*)-*gfp* (*spec*)] was generated by transforming *B. subtilis* BDR3558 with pFR027.

**BDR3632** [Δ*spoVV ycgO::spoVV*(*G96A*)-*gfp* (*spec*)] was generated by transforming *B. subtilis* BDR3558 with pFR024.

**BDR3646** [Δ*gerAB*::*erm* Δ*spoVV ycgO::spoVV-gfp* (*wt*) (*spec*)], **BDR3647** [Δ*gerAB*::*erm* Δ*spoVV ycgO::spoVV*(*G96A*)-*gfp* (*spec*)], **BDR3648** [Δ*gerAB*::*erm* Δ*spoVV ycgO::spoVV*(*N97A*)-*gfp* (*spec*)], **BDR3649** [Δ*gerAB*::*erm* Δ*spoVV ycgO::spoVV*(*F141A*)-*gfp* (*spec*)], **BDR3650** [Δ*gerAB*::*erm* Δ*spoVV ycgO::spoVV*(*F302A*)-*gfp* (*spec*)] and **BDR3651** [Δ*gerAB*::*erm* Δ*spoVV ycgO::spoVV*(*Q310A*)-*gfp* (*spec*)] were generated by transforming *B. subtilis* BDR3527, BDR3632, BDR3562, BDR3615, BDR3563 and BDR3564, respectively, with a PCR product containing Δ*gerAB::erm* (amplified with the oligonucleotide primers oFR1 and oFR2 and gDNA from the strain BAM786 as template).

**BDR3699** (Δ*spoVFB::erm*) and **BDR3700** (Δ*gerAB* Δ*spoVFB::erm*) were generated by direct transformation of *B. subtilis* 168 and BDR3158, respectively, with a PCR product containing the mutation Δ*spoVFB::erm* [amplified with the oligonucleotide primers oFR60 + oFR61 and gDNA of the strain Δ*spoVFB::erm* (BKE collection) as template].

**Plasmids construction**

**pFR001** [*ycgO*::P$_{hyperspank}$-*spoVFAB* (*erm*)] was constructed in a two-way ligation with a *Spe*I-*Sph*I PCR product containing the *spoVFAB* operon (amplified with oligonucleotide primers oDR1247 and oDR1257 and gDNA from *B. subtilis* 168 as template) and pER67 cut with *Spe*I and *Sph*I. pER67 [*ycgO::*P$_{hyperspank}$ (*lacI*) (*erm*)] is a double crossover vector with an IPTG-inducible promoter for ectopic integration at the *ycgO* locus (Rudner Lab stock).

**pFR002** [*amyE*::P$_{xylA}$-*spoVV* (*spec*)] was constructed in a two-way ligation with a *Sal*I-*BamH*I PCR product containing *spoVV* (amplified with oligonucleotide primers oDR1250 and oDR1251 and gDNA from *B. subtilis* 168 as template) and pDR150 cut with *Sal*I and *BamH*I. pDR150 [*amyE::*P$_{xylA}$ (*xylR*) (*spec*)] is a double crossover vector with a xylose inducible promoter for ectopic integration at the *amyE* locus (Rudner Lab stock).

**pFR008** [*ycgO::*P$_{yeeK}$-*optRBS-spoVV-gfp* (*erm*)] was constructed in a three-way ligation with an *EcoR*I-*BamH*I PCR product containing the SigK-responsive *yeeK* promoter (amplified with oligonucleotide primers oFR11 and oFR12 and gDNA from *B. subtilis* 168 as template) and a *Nhe*I-*BamH*I PCR product containing *spoVV* with an optimized RBS fused to the *mGFPmut3a* (amplified with oligonucleotide primers oFR13 and oFR15 and gDNA from BDR3449 as template) into pER61 cut with *EcoR*I and *BamH*I. pER61 (*ycgO*::*erm*) is a double-crossover vector for ectopic integration at the *ycgO* locus (Rudner Lab stock).

**pFR009** [*ycgO::*P$_{spoVV}$-*optRBS-spoVV-gfp* (*erm*)] was constructed in a three-way ligation with a *Hind*III-*Nhe*I PCR product containing the promoter of *spoVV* (amplified with oligonucleotide primers oFR16 and oFR17 and gDNA from *B. subtilis* 168 as template) and a *Nhe*I-*BamH*I PCR product containing *spoVV* with an optimized RBS fused to the *mGFPmut3a* reporter (amplified with oligonucleotide primers oFR13 and oFR15 and gDNA from BDR3449 as template) into pER61 between *Hind*III and *BamH*I sites.

**pFR011** [*ycgO*::*spoVV-gfp* (*spec*)] was constructed in a two-way ligation with a *Spe*I-*BamH*I PCR product containing *spoVV* fused to the *mGFPmut3a* reporter (amplified with oligonucleotide primers oFR23 + oFR24 and gDNA from BDR3449 as template) and pKM83 cut with *Spe*I and *BamH*I. pKM83 (*ycgO*::*spec*) is a double-crossover vector for ectopic integration at the *ycgO* locus (Rudner Lab stock).

**pFR017** [*ycgO*::*spoVV*(*N97A*)-*gfp* (*spec*)] was constructed by site-directed mutagenesis using oligonucleotide primers oFR32 + oFR33 and plasmid pFR011.

**pFR018** [*ycgO*::*spoVV*(*F302A*)-*gfp* (*spec*)] was constructed by site-directed mutagenesis using oligonucleotide primer oFR34 and plasmid pFR011.

**pFR019** [*ycgO*::*spoVV*(*Q310A*)-*gfp* (*spec*)] was constructed by site-directed mutagenesis using oligonucleotide primer oFR36 and plasmid pFR011.

**pFR024** [*ycgO*::*spoVV*(*G96A*)-*gfp* (*spec*)] was constructed by site-directed mutagenesis using oligonucleotide primers oFR40 + oFR41 and plasmid pFR011.

**pFR027** [*ycgO*::*spoVV*(*F141A*)-*gfp* (*spec*)] was constructed by site-directed mutagenesis using oligonucleotide primers oFR46 + oFR47 and plasmid pFR011.
