# Supplementary material for: A two-step transport pathway allows the mother cell to nurture the developing spore in Bacillus subtilis
Source: PLoS Genet. 2017 Sep 25;13(9):e1007015. doi: 10.1371/journal.pgen.1007015 (PMC5629000; doi:10.1371/journal.pgen.1007015)
Supplement: S1 Table — (PDF) [file pgen.1007015.s006.pdf]

Table S1. Strains used in this study.

Strain	Genotype	Source	Figure(s)
168	Wild-type (*trpC2*)	Zeigler *et al*., 2008	1, 3, 5, S1, S2 and S3
BAM786	Δ*gerAB::erm*	Koo *et al.*, 2017	
BDR3158	Δ*gerAB*	Ramírez-Guadiana *et al., 2017*	1, S1 and S2
BDR3151	Δ*spoVFA*	Ramírez-Guadiana *et al., 2017*	1, S1 and S2
BDR3154	Δ*spoVV*	Ramírez-Guadiana *et al., 2017*	1, 5 and S2
BDR3205	Δ*gerAB* Δ*spoVV*	Ramírez-Guadiana *et al., 2017*	1, 3, 5 and S2
BDR3205	Δ*gerAB* Δ*spoVT*	Ramírez-Guadiana *et al., 2017*	1
BDR3206	Δ*gerAB* Δ*spoVFA*	Ramírez-Guadiana *et al., 2017*	1, S1 and S2
BDR3205	Δ*gerAB* Δ*ytaF*	Ramírez-Guadiana *et al., 2017*	1
BDR3312	Δ*spoVV::spec*	Ramírez-Guadiana *et al., 2017*	
BDR3699	Δ*spoVFB*::*erm*	This work	S1
BDR3700	Δ*gerAB* Δ*spoVFB*::*erm*	This work	S1
BDR3414	Δ*spoVV* Δ*gerA::spec*	This work	3
BDR3416	*ycgO::cat*	This work	
BDR3430	*ycgO::* $P_{hyperspank}$-*spoVFAB* (*erm*)	This work	4 and S3
BDR3432	*ycgO::* $P_{hyperspank}$-*spoVFAB* (*erm*) *amyE::*P_{xylA}-*spoVV* (*spec*)	This work	4 and S3
BDR3449	*spoVV-gfp* (*spec*)	This work	
BDR3465	*ycgO::*P_{yeeK}-*optRBS-spoVV-gfp* (*erm*)	This work	
BDR3466	*ycgO::*P_{spoVV}-*optRBS-spoVV-gfp* (*erm*)	This work	
BDR3468	Δ*spoVV ycgO::*P_{yeeK}-*optRBS-spoVV-gfp* (*erm*)	This work	
BDR3469	Δ*spoVV ycgO::*P_{spoVV}-*optRBS-spoVV-gfp* (*erm*)	This work	2
BDR3471	Δ*gerA::spec* Δ*spoVV ycgO::*P_{yeeK}-*optRBS-spoVV-gfp* (*erm*)	This work	3
BDR3458	Δ*spoIIQ::kan*	This work	
BDR3472	Δ*spoIIQ::kan* Δ*spoVV ycgO::*P_{spoVV}-*optRBS-spoVV-gfp* (*erm*)	This work	2
BCR1117	Δ*spoIIIAH*	Laboratory stock	
BDR3474	Δ*spoIIIAH* Δ*spoVV::spec ycgO::*P_{spoVV}-*optRBS-spoVV-gfp* (*erm*)	This work	2
BDR3507	*ycgO::spoVV-gfp* (*spec*)	This work	
BDR3527	Δ*spoVV ycgO::spoVV-gfp* (*spec*)	This work	5
BDR3558	Δ*spoVV ycgO::cat*	This work	
BDR3632	Δ*spoVV ycgO::spoVV-gfp* (*G96A*) (*spec*)	This work	5
BDR3562	Δ*spoVV ycgO::spoVV-gfp* (*N97A*) (*spec*)	This work	5
BDR3615	Δ*spoVV ycgO::spoVV-gfp* (*F141A*) (*spec*)	This work	5
BDR3563	Δ*spoVV ycgO::spoVV-gfp* (*F302A*) (*spec*)	This work	5
BDR3564	Δ*spoVV ycgO::spoVV-gfp* (*Q310A*) (*spec*)	This work	5
BDR3646	Δ*gerAB::erm* Δ*spoVV ycgO*::*spoVV-gfp* (wt) (*spec*)	This work	5
BDR3647	Δ*gerAB::erm* Δ*spoVV ycgO*::*spoVV-gfp* (G96A) (*spec*)	This work	5
BDR3648	Δ*gerAB::erm* Δ*spoVV ycgO*::*spoVV-gfp* (N97A) (*spec*)	This work	5
BDR3649	Δ*gerAB::erm* Δ*spoVV ycgO*::*spoVV-gfp* (F141A) (*spec*)	This work	5
BDR3650	Δ*gerAB::erm* Δ*spoVV ycgO*::*spoVV-gfp* (F302A) (*spec*)	This work	5
BDR3651	Δ*gerAB::erm* Δ*spoVV ycgO*::*spoVV-gfp* (Q310A) (*spec*)	This work	5

All unmarked mutants are in-frame deletions generated by Cre-mediated recombination and contain a *lox72* scar.
