# Supplementary material for: A two-step transport pathway allows the mother cell to nurture the developing spore in Bacillus subtilis
Source: PLoS Genet. 2017 Sep 25;13(9):e1007015. doi: 10.1371/journal.pgen.1007015 (PMC5629000; doi:10.1371/journal.pgen.1007015)
Supplement: S2 Table — (PDF) [file pgen.1007015.s007.pdf]

Table S2. Plasmids used in this study.

Plasmid	Description	Source
pFR001	*ycgO*::$P_{hyperspank}$-*spoVFAB* (*erm*) (*amp*)	This work
pFR002	*amyE*::P_{xylA}-*spoVV* (*spec*) (*amp*)	This work
pFR008	*ycgO::*P_{yeeK}-*optRBS-spoVV-gfp* (*erm*) (*amp*)	This work
pFR009	*ycgO::*P_{spoVV}-*optRBS-spoVV-gfp* (*erm*) (*amp*)	This work
pFR011	*ycgO*::*spoVV-gfp* (*spec*) (*amp*)	This work
pFR017	*ycgO*::*spoVV*(N97A)-*gfp* (*spec*) (*amp*)	This work
pFR018	*ycgO*::*spoVV*(F302A)-*gfp* (*spec*) (*amp*)	This work
pFR019	*ycgO*::*spoVV*(Q310A)-*gfp* (*spec*) (*amp*)	This work
pFR024	*ycgO*::*spoVV*(QG96A)-*gfp* (*spec*) (*amp*)	This work
pFR027	*ycgO*::*spoVV*(F141A)-*gfp* (*spec*) (*amp*)	This work
