# Supplementary material for: A two-step transport pathway allows the mother cell to nurture the developing spore in Bacillus subtilis
Source: PLoS Genet. 2017 Sep 25;13(9):e1007015. doi: 10.1371/journal.pgen.1007015 (PMC5629000; doi:10.1371/journal.pgen.1007015)
Supplement: S3 Table — (PDF) [file pgen.1007015.s008.pdf]

Table S3. Oligonucleotide primers used in this study.

Primer	Sequence
oDR1247	ggACTAGTcaaagaaggtgaacgtttagaatg
oDR1257	gccGCATGCtagaatatgcgggtgatgaccg
oDR1250	gccGTCGACagcatcggggaggtaagaatg
oDR1251	cgcGGATCCgccggaagtagagttggcgc
oDR1262	ggcggcttcgttcttgctatg
oDR1263	tttacttccggaaccctcgagtcctttttttgttccgtttgtcag
oDR1264	ctcgagggttccggaagtaaa
oDR1265	gttccatccgattatcacggg
oDR1266	cccgtgataatcggatggaacggatgagtcaaaaccctcatcc
oDR1267	agtggtgtggaacatgagagt
oFR1	cttgtttccttccatcaggg
oFR2	ttctgacctcgtttcccagc
oFR3	agggcatggttgtgtatctgc
oFR5	tgaatggtttctttattaggc
oFR8	gtttcgcctcagggtatatg
oFR11	gccGAATTCctgtaatcggtgtggaaggc
oFR12	gccGCTAGCcataggttacagtccagagt
oFR13	gccGCTAGCacataaggaggaactactatgaacttgtcgaagattaataca
oFR15	gccGGATCCttatttgtatagttcatccatgccatgtgt
oFR16	gccAAGCTTagcatgcacaagctgtctgac
oFR17	gccGCTAGCtgattctttgtactaatatacgagcatagg
oFR23	ggcACTAGTacaccgagatgagcaagccct
oFR24	gccGGATCCttatttgtatagttcatccatgccatgtgt
oFR32	atgggaatggcatcaggagccccagcgggcgcaaaactcac
oFR33	gtgagttttgcgcccgctggggctcctgatgccattcccat
oFR34	gtcagctttattcttggggcaagcggcttttccgtacaagc
oFR36	ggcttttccgtacaagctgcagtggcaggtattttatcgga
oFR40	gctatgggaatggcatcagcgaacccagcgggcgcaaaact
oFR41	agttttgcgcccgctgggttcgctgatgccattcccatagc
oFR46	ggcgctgttgccgtcggtgcgtttcaaaacgcatcactagg
oFR47	cctagtgatgcgttttgaaacgcaccgacggcaacagcgcc
oFR60	ggtcatgacattcgtgtccc
oFR61	gctgctggcttggatcatcc
oKO260	cgcgtacgctgcatatgtcta
oKO0	ctcgttcatagtagttcctcc

Capital letters indicate the recognition sequence for restriction enzymes and underlines indicate mutated bases.
